# Motor performance after treatment of pilocytic astrocytoma in the posterior fossa in childhood

**DOI:** 10.1002/cnr2.1548

**Published:** 2021-10-12

**Authors:** Ingela Kristiansen, Gunilla E. Frykberg, Anette Höglund, Annette Sondell, Bo Strömberg, Per Frisk

**Affiliations:** ^1^ Department of Women's and Children's Health Uppsala University and Uppsala University Children's Hospital Uppsala Sweden; ^2^ Department of Neuroscience/Rehabilitation Medicine Uppsala University Uppsala Sweden

**Keywords:** ADL, balance, childhood, motor performance, pilocytic astrocytoma, posterior fossa

## Abstract

**Background:**

Pilocytic astrocytoma is the most common brain tumour type in childhood located in the posterior fossa, and treated mainly with surgery. These tumours have low mortality, but knowledge concerning its long‐term outcome is sparse.

**Aims:**

The aim was to investigate if patients treated for pilocytic astrocytoma in the posterior fossa had motor complications, including balance, motor and process skills.

**Methods and Results:**

This descriptive single‐centre study includes eight children and 12 adults, treated for pilocytic astrocytoma as children. Motor performance was investigated with Bruininks–Oseretsky Test of Motor Proficiency, Second Edition, and dynamic balance with the mini‐balance evaluation systems test. Physiological cost index, six‐minute walk test, hand grip strength and assessment of motor and process skills were also evaluated. Ten patients reported motor difficulties, mainly from the upper limbs. The motor performance test showed results within normal limits except for manual dexterity, which was significantly below mean (*p* = .008). In the dynamic balance test patients had significantly lower results compared with controls (*p* = .036). Physiological cost index, six‐minute walk tests and hand grip strength showed results within normal limits. In the Assessment of Motor and Process Skills, patients over 16 years had significantly lower results compared with test norms for motor activities of daily living (ADL) and 30% of all patients scored below the cut‐off level for difficulties with motor skills.

**Conclusions:**

Motor performance for patients treated for pilocytic astrocytoma in the posterior fossa in childhood is satisfactory but some patients display difficulties with balance, manual dexterity and ADL motor skills. Thus, it is important to identify those in need of motor follow‐up and training.

## 1 INTRODUCTION

The yearly incidence of brain tumours in children younger than 15 years in Sweden 1984–2005 was 4.2/100 000. Survival rates have improved, but vary across different tumour types.^1^ There is also variation regarding medical, cognitive and psychological long‐term complications, caused either by the tumour itself and/or by the treatment.^2^ Complications are mainly reported among children treated for high‐grade tumours,^3^ but in an earlier study, we found complications also among children treated for low‐grade tumours.^4^

Pilocytic astrocytoma is the most common brain tumour type in childhood located in the posterior fossa, and treated mainly with surgery. These tumours have a low mortality and favourable long‐term outcome.^5^, ^6^ Although there are studies describing neurological, cognitive and behavioural complications among these patients, there is still a lack of knowledge concerning the extent of late effects.^7^, ^8^, ^9^, ^10^, ^11^ In a study by our group, we found favourable clinical outcome, but 40% reported learning difficulties.^12^

The cerebellum plays important roles in the acquisition of motor skills. This involves development from controlled to automatic processing, where movements that initially require problem solving and attention become increasingly more efficient and require less attention.^13^ Diseases involving the cerebellum can lead to ataxia, which is characterised by incoordination of balance, gait, extremity and eye movements and dysarthria.^14^ When assessing conditions involving the cerebellum, the concepts of postural control and balance are important. Postural control is defined as the act of maintaining, achieving or restoring a state of balance during any posture or activity.^15^ Balance is defined as the ability to maintain the body's centre of mass over its base of support, and is a composite ability involving integration of information from several sensory systems.^16^ Like any other motor skill, postural control strategies can become more efficient with training and practice.^15^

Motor consequences have not been as extensively evaluated as cognition in studies of children treated for brain tumours.^17^, ^18^ However, in a study by Piscione survivors of posterior fossa tumours demonstrated decreased physical functioning^17^ and in another study by Rueckriegel, results showed impairment of fine motor function.^19^

Against this background, more knowledge is needed about the type and extent of motor complications in patients treated for pilocytic astrocytoma in the posterior fossa. Our hypothesis was that these patients had affected motor performance. Therefore, our aims were to investigate whether patients had self‐perceived difficulties with motor performance. We also wanted to investigate how they performed in tests of motor proficiency, including balance and if they had diminished gait efficiency, affected functional exercise capacity and reduced grip strength. Lastly, we wanted to investigate whether the patients had affected motor and process activities of daily living (ADL) skills, and if the patients with motor difficulties also reported difficulties in school.

## 2 MATERIAL AND METHODS

### 2.1 Participants

This single‐centre study was performed 2015–2017 at Uppsala University Children's Hospital, Sweden, a tertiary referral centre for children with tumours in the central nervous system (CNS), serving six counties in mid‐Sweden with a population of 1.7 million people. Patients were retrieved from the local and the National Brain Tumour Registry. A total of 27 patients <18 years of age with a low‐grade astrocytoma in the posterior fossa diagnosed and treated in childhood between 1995 and 2011 were identified. At the time of this investigation, nine were children (9–17 years) and 18 adults (21–33 years). Patients were included at least 5 years after end of treatment. This study includes the same patient group as a former study from our research group,^12^ except two adults who only participated in telephone interviews. Three patients did not answer several invitations, and two declined participation. Thus, 20 patients agreed to take part (12 adults and eight children; 74%). The mean age at tumour presentation was 8.3 years (standard deviation [SD] 4.3), and the mean age at participation in the study was 20.2 (SD 7.3) years. Mean time from diagnosis to participation was 12.2 (SD 4.6) years.

The included patients showed normal psychomotor development prior to diagnosis, except for one patient, diagnosed at 1 year of age with a delayed psychomotor development. All patients were treated surgically, and in 17 patients, the operation was considered as a complete resection. Three patients had a remaining tumour and were re‐operated shortly after the initial operation. Another three patients relapsed; one was treated with re‐surgery, one with re‐surgery and chemotherapy (initially vincristine and carboplatin, later changed to vinblastine because of hearing loss) and one with gamma knife radio surgery. None had a metastatic tumour.

Nine patients had contact with a physiotherapist immediately after treatment.

### 2.2 Procedure

Upon acceptance, participants were invited to the Folke Bernadotte Regional Rehabilitation Center in Uppsala to undergo investigations performed by a multi‐professional team, 2 days for adults and 3 days for children. A schedule was made for the activities during the days, including a lunch break and rest between the different tests. The investigations included an interview, a neurological examination, tests of motor performance and assessment of motor and process skills.

#### 2.2.1 Tests of motor performance

##### Bruininks–Oseretsky Test of Motor Proficiency, Second Edition

The Bruininks–Oseretsky Test of Motor Proficiency, Second Edition (BOT‐2) measures motor skills in individuals aged four through 21 years.^20^ It assesses proficiency in four motor‐area composites using eight subtests: Fine manual control (fine motor precision and fine motor integration), manual coordination (manual dexterity and upper‐limb coordination), body coordination (bilateral coordination and balance) and strength and agility (running speed and agility and strength). Results were calculated into scaled scores, mean (M) 15, SD 5 with American norms.

##### Physiological cost index

Measurements of energy expenditure are often used to quantify gait efficiency by using heart rate recordings.^21^ These provide an estimated measure of energy expenditure, based on a linear relationship between heart rate and oxygen uptake at a sub‐maximal activity level. Physiological cost index (PCI) is calculated as a quotient of the difference between work and rest heart rate divided by the walking speed, expressed as heartbeats per metre:

$$PCI\;(\text{beats}/\text{metre})=\frac{\text{work}\;\text{heart}\;\text{rate}\;–\;\text{rest}\;\text{heart}\;\text{rate}\;(\text{beats}/\text{min})}{\text{walking}\;\text{speed}\;(\text{metre}/\text{min})}\;$$

The patients walked 375 m indoors at a self‐selected speed. Directly after the test, the exertional effort was assessed according to the Borg rating of perceived exertion (RPE) scale where the lowest level six, corresponds with no exertion and the highest 20, with maximal exertion.^22^ The reference values (M 0.44, SD 0.13) were taken from a study by Bratteby Tollerz,^21^ which included 20 healthy children aged 5–16 years. These values have been used for all patients, based on the assumption that energy cost will be approximately the same for all ages, because rest and work heart rate decreases with age and walking speed increases.^21^

##### Six‐minute walk test

The six‐minute walk test (6MWT) is a method to assess the submaximal level of functional exercise capacity. The test measures the distance in metres the patient can walk on a flat, hard surface in a period of 6 min.^23^ The level of exertion during the test was assessed by the RPE scale. Reference values, divided into age groups and sex, were taken from a study by Geiger^23^ including 528 children.

##### Hand grip strength

Grip strength is routinely assessed to evaluate upper extremity impairments, strength changes and work capacity. It provides information about hand function^24^ and is an indicator of general health.^24^ A three Jamar hydraulic hand‐held dynamometer (Samons Preston Rolyan, Bolingbrook, IL) was used to measure grip strength in pounds (lb). Normative data, divided into sex and age groups, were collected from a study by McQuiddy,^24^ where 1508 students aged 6–19 years participated, and from a study by Fain,^25^ with 237 participants, aged 20–34 years.

##### Mini‐balance evaluation systems test

The mini‐balance evaluation systems test (Mini‐BESTest) is a clinical balance scale including 14 items that examine motor performance tasks related to dynamic balance, such as dynamic body stability, transfers, gait, variation of support surfaces and visual conditions, obstacle negotiation, reaction to external forces and performance during dual tasking with cognitive challenge.^26^ The administration time is about 15 min, which is an advantage over more extensive instruments, and is used and validated to assess balance impairments in several conditions, including neurological diseases.^26^ In this study, we performed a modified variant, where half of the subtests were recorded three times with simultaneous sensor data collection for further analyses. This routine with repeated registrations is customary when collecting time series data with sensors. Thus, the collection of the Mini‐BESTest data in this study took longer time (approximately 45 min) than the usual 15 min. The Mini‐BESTest has been translated into Swedish and validated for Parkinson's disease and stroke.^27^ In the original publication, 28‐point summated scores were used, and only the most affected side for items of one‐leg stance and lateral compensatory stepping were included. However, when comparing the effects of using a 32‐point or a 28‐point scoring scale, the effects on the results were minimal.^28^ We used the Swedish version of the test, where the 32‐point scoring scale was used. In a study by O'Hoski normative values were presented for the ages 50–89 years.^16^ We have no knowledge of any established normative values for children and young adults. Therefore, a control group consisting of two sex‐, age‐, length‐ and weight‐matched healthy controls for each patient was recruited (22 adults and 16 children) for comparison. One adult patient was excluded from participation in the Mini‐BESTest due to a bone‐length difference that influenced balance, hence controls were not recruited for this patient.

#### 2.2.2 Test of performance of activities of daily living

##### Assessment of motor and process skills

The assessment of motor and processing skills (AMPS) is an observational evaluation of performance of ADL^29^, ^30^ and is designed to measure a person's quality of performance of ADL tasks. It evaluates two domains: the quality of ADL motor and ADL process tasks performance. A minimum of two ADL tasks are selected from over 120 standardised ADL tasks and assessed for 16 motor and 20 process skills. Raw scores are converted into logits (log‐odds probability units) by the AMPS computer‐scoring software to compare AMPS motor and process logits measures with results in the AMPS database.^29^, ^31^ Mean ADL motor and ADL process ability measures for typically developed children and adults stored in the database can provide a reference for interpreting the results of an AMPS evaluation. For children aged 9–15 years the measures for ADL motor ability is M 2.10, SD 0.47, and for adolescents and adults 16–59 years M 2.88, SD 0.51. The measures for ADL process ability are for children 9–15 years M 1.13, SD 0.44, and for adolescents and adults 16–59 years M 1.20, SD 0.46. The AMPS computer program provides a graphic report of the results for each patient. A result below two logits for ADL motor and below one logit for ADL process skills indicates diminished quality and performance of ADL activities.^30^

## 3 STATISTICS

Statistics were calculated using the SPSS statistical program, version 26. Due to the small sample size and unknown distribution, we consistently used non‐parametric tests. One sample Wilcoxon signed rank test was used to compare means with normative data. The Mann Whitney *U* test was used to compare the patients with the control group in the Mini‐BESTest and concerning age, weight and length. The level of significance was set at *p* < .05. As this was an exploratory study, we did not adjust for multiple comparisons, and *p*‐values were interpreted with caution.

## 4 RESULTS

### 4.1 Reported motor performance

Fourteen participants, five children and nine adults reported that they had motor symptoms postoperatively, mainly difficulties with balance and stiffness in the neck. In the study interviews, 10 participants, four children and six adults, reported that they had motor difficulties that affected their motor performance to some extent (Table 1). Six had difficulties affecting the upper limbs and two affecting balance, while one experienced one‐sided muscular weakness. One patient had a pronounced difference in leg length present before the tumour diagnosis.

**TABLE 1:** Motor symptoms postoperatively and reported in the study interviews

	Postoperative symptoms			Symptoms reported in the study interviews		
	Number	Children	Adults	Number	Children	Adults
Upper limb						
Slow movements in the left hand				1		1
Tremor in the hands during work				1		1
Increased fatigability in the left hand				1	1	
Less function in the left arm	1	1		1	1	
Tremor in the left hand				1	1	
Changed handedness from right to left	1		1	1		1
General motor and balance difficulties						
Stiff neck and back	4	2	2			
Balance problems	5	1	4	1		1
Tremor in the left side and balance difficulties	1		1	1		1
Muscular weakness in the left side	1	1		1	1	
Leg length difference	1		1	1		1
Total	14	5	9	10	4	6

### 4.2 BOT‐2

All 20 participants performed BOT‐2 (Table 2), The results were within normal limits compared with norms, except for manual dexterity, which was significantly below mean (*p* = .008), and a tendency towards low results in the subtest balance (*p* = .051). Nine patients had a result below −1 SD in balance and seven in manual dexterity.

**TABLE 2:** Results of BOT‐2 for all, children and adults (reference value of the test M 15, SD 5)

	Number	Mean	SD	Median	Minimum	Maximum	*p*‐values
Bilateral coordination							
All	20	15.2	4.5	17.0	6	20	.625
Children	8	16.4	4.1	17.5	9	20	
Adults	12	14.4	4.7	17.0	6	18	
Balance							
All	20	12.1	6.1	11.5	4	22	.051
Children	8	11,6	6.9	10.0	4	22	
Adults	12	12.3	5.8	12.0	4	21	
Running speed and agility							
All	20	16.5	4.9	17.5	4	25	.095
Children	8	16.9	4.4	18.0	8	21	
Adults	12	16.3	5.4	17.0	4	25	
Strength							
All	20	14.6	4.2	14.5	7	24	.569
Children	8	14.9	3.1	15.0	9	20	
Adults	12	14.3	4.9	13.5	7	24	
Fine motor precision							
All	20	16.6	4.1	19.0	10	23	.148
Children	8	17.7	3.9	18.0	11	23	
Adults	12	15.7	4.1	19.0	10	19	
Fine motor integration							
All	20	14.6	5.0	13.5	7	23	.707
Children	8	14.7	5.4	13.0	7	23	
Adults	12	14.6	5.0	16.5	7	20	
Manual dexterity							
All	20	12.0	4.1	11.5	6	22	.008
Children	8	11.9	3.7	12.5	6	17	.049
Adults	12	12.1	4.6	11.0	8	22	.083
Upper‐limb coordination							
All	20	15.8	3.8	16.5	7	21	.320
Children	8	13.6	4.4	14.0	7	20	
Adults	12	17.2	2.8	18.0	11	21	

*Note*: *p*‐values are presented separately for children and adults when significant for the whole population.

Abbreviation: BOT‐2, Bruininks–Oseretsky Test of Motor Proficiency, Second Edition.

### 4.3 PCI

The patients had results within normal limits compared with the reference values (M 0.43, SD 0.14), and rated the exertion as very light.

### 4.4 6MWT

All results were within normal limits compared with norms. Female participants scored exertion as somewhat hard, while males 12–15 years scored exertion as very light and those above 16 years as light.

### 4.5 Hand grip strength

All results were within normal limits compared with the age norms.

### 4.6 Mini‐BESTest

Twenty‐four children (eight patients and 16 controls) and 33 adults (11 patients and 22 controls) performed the Mini‐BESTest (Table 3). All participants could perform the test properly and understood the test instructions. There were no significant differences between the patients and controls concerning age, length and weight (*p* = .857, *p* = .884 and *p* = .657, respectively). The patients had significantly lower results compared with the controls for the whole sample (*p* = .036) and for the children (*p* = .016) (Table 3).

**TABLE 3:** Results for the Mini‐BESTest and comparison between patients and controls

	Number	Mean	SD	Median	Minimum	Maximum	*p*‐values
All	57	29.4	2.6	30.0	19	32	.036
Children	24	28.5	2.5	29.0	19	31	.016
Adults	33	30.0	2.5	31.0	21	32	.317
Patients	19	28.1	3.6	28.0	19	32	
Children	8	26.9	3.4	27.5	19	30	
Adults	11	29.0	3.6	30.0	21	32	
Controls	38	30.0	1.7	31.0	26	32	
Children	16	29.3	1.4	29.5	26	31	
Adults	22	30.5	1.6	31.0	26	32	

Abbreviation: Mini‐BESTest, the mini‐balance evaluation systems test.

### 4.7 AMPS

All patients performed AMPS (Table 4). Examples of assessed tasks included making omelettes, baking muffins or brownies, making a fresh fruit salad, or an open‐face meat or cheese sandwich with sliced vegetables. Children 9–15 years old had results within normal limits compared with norms for both motor and process ADL. Four had results below the cut‐off level of two logits for motor ADL, and one below cut off for process ADL. Patients over 16 years had significantly lower results for motor ADL compared with norms (*p* = .01) and significantly higher for process ADL (*p* = .023). Two patients had results below the cut off level for motor and one for process ADL.

**TABLE 4:** Results of AMPS in logits

Age in years	*N*	Mean	Test reference M	SD	Test reference SD	Median	Min	Max	*p*‐values	Number below the cut‐off level
Motor ADL										
9–15	7	1.8	2.10	0.35	0.47	1.8	1.33	2.18	.108	4
16–	13	2.4	2.88	0.32	0.51	2.5	1.7	2.6	.01	2
Process ADL										
9–15	7	1.2	1.13	0.5	0.44	1.1	0.38	1.99	.735	1
16–	13	1.5	1.20	0.4	0.46	1.4	0.38	2.05	.023	1

Abbreviations: ADL, activities of daily living; AMPS, assessment of motor and processing skills.

## 5 DISCUSSION

In the present study, we investigated motor performance and the ability to perform ADL tasks in a group of children and adults treated for pilocytic astrocytoma in the posterior fossa as children. The follow‐up time for the included patients is long, and the performed investigations include assessments that provide a broad picture of possible motor strengths and difficulties. Our inclusion of AMPS means that we also have performed a qualitative assessment of performance of ADL skills.

Patients treated for this tumour have a favourable clinical outcome, although difficulties with motor performance were reported in the interviews. The results of BOT‐2 show that the patients had significantly low results in manual dexterity and 45% of the patients had results in the lower range in balance. The patients had significantly lower results compared with controls in the Mini‐BESTest. Results of PCI, 6MWT and hand grip strength showed no indications of decreased physical function.

The patients' results of AMPS were average, except that 30% had results under the cut‐off level for difficulties in motor ADL. Patients over 16 years had significantly lower results for motor ADL. This indicates motor difficulties in performing ADL tasks of importance in everyday life.

The findings with significantly lower results in manual dexterity and affected balance are in accordance with results in other studies, where children treated for cerebellar tumours were investigated.^17^, ^19^ In the study by Piscione, patients treated for pilocytic astrocytoma had results in the low normal range in BOT‐2 for body coordination (where the subtest balance is included).^17^ Rueckriegel^19^ investigated fine motor function by using a digitalizing graphic tablet to study kinematic parameters (speed, automation, variability and pressure) of different movement complexity levels. The results showed impairment of fine motor function, especially in patients treated for medulloblastoma. Patients treated for pilocytic astrocytoma showed more subtle impairment in the complex hand movements of handwriting. The subtest Manual dexterity in BOT‐2 includes similar complex activities important in daily life, for example holding and using eating utensils, buttoning buttons, handwriting and recreational activities such as playing cards and putting together puzzles.^20^

In a review Turner^32^ stated that balance abilities in survivors of acute lymphatic leukaemia and CNS tumours are decreased when compared with healthy controls. Few studies included investigations of dynamic balance and the authors suggest that dynamic balance should be evaluated since this is more clearly related to functional tasks. We evaluated dynamic balance with the Mini‐BESTest, in this homogenous group of patients treated for pilocytic astrocytoma in the posterior fossa together with a control group for comparison. We found a significant difference between all patients and controls and also between the children and their controls. This indicates that the patients had difficulties with functional balance tasks of potential importance in daily life.

Küper et al.^33^ performed a longitudinal study of the restoration of function after cerebellar tumour removal and found a substantial improvement of motor function during the first year after surgery. They argue that due to the fact that maturation of the cerebellum is still ongoing during childhood and adolescence, functional recovery may differ depending on age at injury. However, they state that the lesion site and not age‐at‐surgery is critical for motor recovery. Impairments in balance and upper‐limb function were linked to lesions of the inferior vermis and the deep cerebellar nuclei.^33^ In our study, both children and adults reported self‐perceived difficulties with motor functions and had affected test results in balance or manual dexterity. In this cross‐sectional study with a small sample, we were not able to confirm either that the patients' functions were improved over time or the effect of tumour location. Longitudinal studies would be of interest to investigate if restoration continues over a longer time frame, including the possible effect of tumour localisation.

In the interviews, eight patients reported unmet learning difficulties in school, five of whom reported motor difficulties. Rueckriegel^19^ reported a significant association between IQ scores, especially performance IQ and fine motor function. It was not possible to decide if this association was caused by a direct negative influence of fine motor impairment on the test methodology or a coincidence of coexisting fine motor‐ and cognitive deterioration. However, the author states that these findings are in line with other studies.^19^ Thus, it is important to identify and support those with the combined burden of both learning and motor difficulties. Lönnerblad performed a study on the performance of children treated for brain tumours in practical subjects including physical education. The results showed that children treated for brain tumours had significantly lower grades compared with controls,^34^ without any significant impact of tumour grade. This underscores that also children treated for low‐grade tumours are at risk for lower results in physical education. Exercise training has proven to improve physical functioning, mood and cognitive performance,^35^ which also has been shown in children treated with radiation.^35^, ^36^ Although patients treated for pilocytic astrocytoma have a lesser degree of motor and cognitive impairments, some still have difficulties in these domains and might benefit from physical training.

Piscione^17^ underscores the need for physical activity programs for children treated for tumours in the posterior fossa. Nine of our patients had contact with a physiotherapist soon after the end of treatment, although they did not take part in any formal training program during any extensive period. This indicates that at least some of the patients received training of motor skills, but that further training opportunities are sparse and/or that it can be hard to maintain the motivation for training over time.

The main limitation of this study is the small number of patients, which means that the study may lack enough power to reveal all potential differences between the results in the studied group and population norms. Thus, there is a risk that motor difficulties among our patients may be underestimated. Because this is a cross‐sectional study with a small sample size, we have not performed investigations to link observed motor difficulties with tumour size and anatomical location in the posterior fossa.

The strength of the study is that it was possible to identify all 27 children and adults diagnosed with pilocytic astrocytoma in the posterior fossa during childhood in a tertiary referral centre during a defined time period and to follow up 74% of them. This entails a low risk for selection bias. Another strength of the study is the long follow‐up period, and the fact that we have had personal contact with all participants.

## 6 CONCLUSIONS

The long‐term functional outcome for children treated for pilocytic astrocytoma in the posterior fossa is favourable. However, some patients have difficulties with motor performance, especially balance, manual dexterity and motor ADL. This may lead to challenges in daily life, especially among those who also have learning difficulties. Therefore, it is imperative to identify those in need of more thorough motor and cognitive follow‐up programs, including interventions in school. Moreover, there is a need to evaluate the effect of motor training among these patients and to establish appropriate collaboration between paediatric neuro‐oncology clinics and the educational system.

## CONFLICT OF INTEREST

The authors have stated explicitly that there are no conflicts of interest in connection with this article.

## AUTHOR CONTRIBUTIONS

All authors had full access to the data in the study and take responsibility for the integrity of the data and the accuracy of the data. *Conceptualization*, I.K., G.F., A.H., A.S., B.S., P.F.; *Data Curation*, I.K., G.F., A.H., A.S., B.S., P.F.; *Formal Analysis*, I.K., G.F., A.H., A.S., B.S., P.F.; *Funding Acquisition*, I.K., B.S., P.F.; *Investigation*, I.K., G.F., A.H., A.S., B.S., P.F.; *Methodology*, I.K., G.F., A.H., A.S., B.S., P.F.; *Project Administration*, I.K., G.F., A.H., A.S., B.S., P.F.; *Resources*, I.K., G.F., B.S., P.F.; *Software*, I.K., G.F., B.S., P.F.; *Supervision*, I.K., G.F., B.S., P.F.; *Validation*, I.K., G.F., A.H., A.S., B.S., P.F.; *Visualisation*, I.K., G.F., A.H., B.S., P.F.; *Writing – Original Draft*, I.K., G.F., A.H., A.S., B.S., P.F.; *Writing – Review and Editing*, I.K., G.F., A.H., A.S., B.S., P.F.

## ETHICAL STATEMENT

The study was approved by the Regional Ethical Board (EPN Uppsala Log. No. 2015/107). Informed consent was obtained from all participants included in the study.

## Data Availability

The data that support the findings of this study are available on request from the corresponding author. The data are not publicly available due to privacy or ethical restrictions.
